# Radiation Oncology Health Disparities in Pakistan

**DOI:** 10.1200/GO.23.00199

**Published:** 2023-12-21

**Authors:** Muhammad Mohsin Fareed, Muhammad Yahya Hameed, Eileen Samuel

**Affiliations:** ^1^ Department of Radiation Oncology, West Virginia University Cancer Institute, Morgantown, WV; ^2^ University of Arkansas for Medical Sciences, Little Rock, AR; ^3^ Department of Internal Medicine, Nassau University Medical Center, East Meadow, NY

## Abstract

This article discusses problems and potential solutions for improving radiation oncology care in Pakistan.

## Introduction

Social determinants of health are the inter-related factors that play a critical role in cancer care health disparities.^1^ Indigent socioeconomic status is the most important social determinant^2^ as it is associated with high mortality rates and adverse survival outcomes because of late presentation, more aggressive tumor biology, and higher risk of other comorbidities.^3-5^ In 2020, Pakistan was categorized as a lower-middle–income country in South Asia.^6^ Pakistan's annual cancer incidence is 0.17 million,^7^ however, its total expenditure on health as a percentage of gross domestic product (GDP) was only 2.6% in 2014,^8^ which is alarmingly low as compared with other developed nations like United States that spent a GDP of 17.1% on health for the same year.^9^

## Current State of Health Disparity in Pakistan Radiation Oncology Service

**FIG 1:**
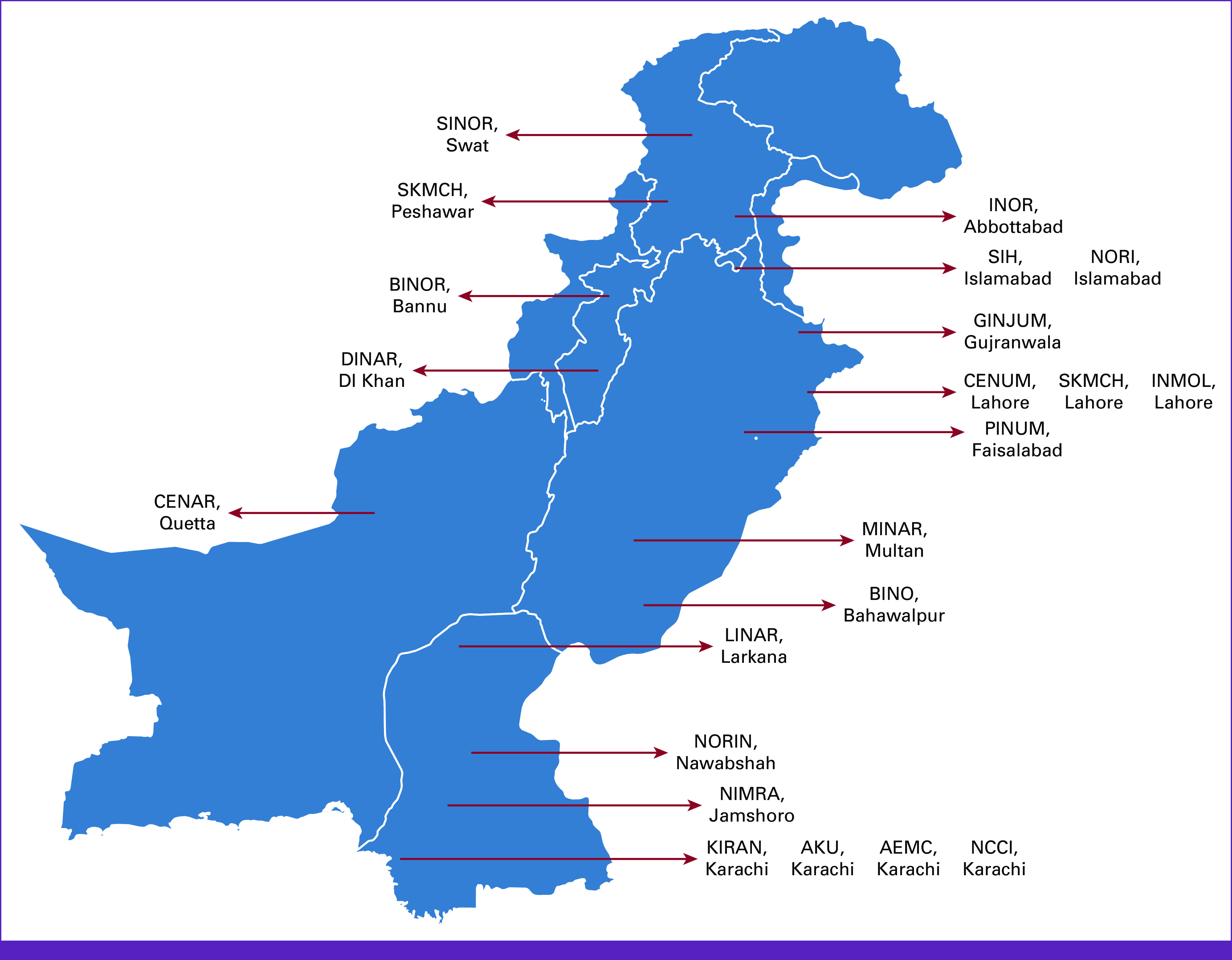
Map of major teaching radiotherapy centers in Pakistan.

**FIG 2:**
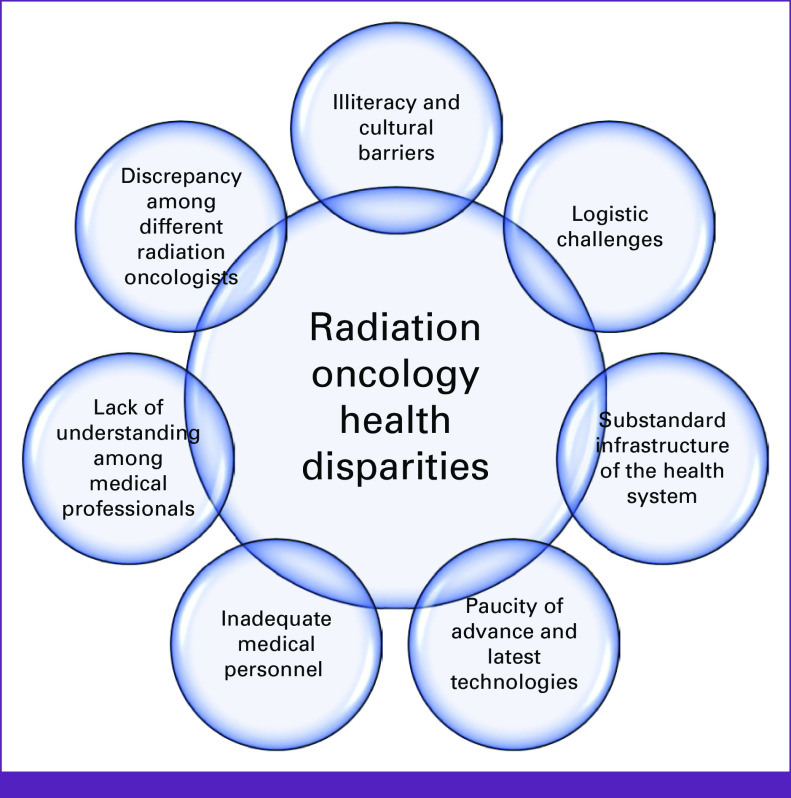
Potential reasons for radiation oncology health disparities in Pakistan.

In the field of radiation oncology, Pakistan is facing multiple challenges. About 60% of the patients diagnosed with cancer may require radiotherapy during their course of treatment; however, only 21.4% of the Pakistani population has access to radiation oncology facilities.^10,11^ Currently, there are 37 hospitals providing radiation oncology services and 260 board-certified oncologists (accredited by the College of Physicians and Surgeons, Pakistan [CPSP]).^12^ Among those, only 1.5% are females.^13^ A map of major teaching radiotherapy centers in Pakistan is shown in Figure 1. The situation remains challenging as the constitutional responsibility of health lies with the federal government and includes regulatory aspect of human resources, health information, global health matters, and drug regulatory policy without gross root-level involvement. Potential reasons and barriers for health disparities and inequity in Pakistan are summarized in Figure 2.

## Factors Affecting Disparities

### Implicit Bias, Illiteracy, and Cultural Barrier

In Pakistan, 64% of the population lives in rural areas with an illiteracy rate of 43%^14,15^ who connect with quacks and faith healers for a significant amount of time,^16^ not only delaying presentation to a valid health system but also declining the compliance rate for treatment. Low literacy rate contributes to poor understanding of their disease (etiology, biology, risk factors, and investigations required for diagnosis) and treatment plan, thus leading to high reluctance rate for treatment.

Apart from overcoming language barriers, cultural barriers are more overwhelming and difficult to handle and have adverse consequences. For instance, female patients with breast cancer not only prefer to be examined by female doctors but also demand female radiation therapists on machine for treatment delivery. As there is a great discrepancy among the number of female radiation workers and number of female patients with cancer,^12^ this not only causes reduced patient comfort but also affects patient position reproducibility, resulting in not only delays in treatment but eventually refusal of treatment delivery. This scenario also applies to patients with gynecologic malignancies undergoing brachytherapy by male doctors. To reduce the anxiety of patients, increased sedation dose and frequency are required. Female patients are either reluctant to discuss the procedure with male doctors or unable to follow the postprocedural instructions like vaginal dilatation. Above all, if they have any complaints before and after the brachytherapy, they prefer to speak to a female paramedical staff instead of talking to a male doctor. This lack of communication increases the risk of complications especially vaginal stenosis, which further affects patient's personal and sexual life.

Another example of sociocultural barrier is evident in the form of about two-thirds of the Pakistanis living in the rural communities without access to healthy living conditions. Inequality in infrastructure and lack of civic responsibility prevent most people from seeking medical attention in these fragile regions. This leads to minimal awareness of cancer screening options and diagnoses, making the overall treatment process stressful. Ultimately, patients accumulate in a nationwide chronic disease burden as they are beyond cure when they do manage to seek care, causing further financial toxicity.^17^

### Geographic and Logistic Challenges

Pakistan is geographically a large entity, being the fifth most populated nation in the world.^6^ Studies show that distance from the radiation center is a well-known risk factor for abysmal radiation care.^18,19^ This leads eventually to disparities in treatment delivery, which include treatment delays, breaks, and incompletion and, as a result, affect the overall survival rates.^20,21^ Patients face logistic challenges because of paucity of radiotherapy facilities. No evidence is currently endowed with outcomes of patients with cancer facing challenges of traveling in Pakistan. However, it can be envisioned by glancing at the transport infrastructure of Pakistan, which is primarily dependent on roads that make up 90% of national passenger traffic and hence, causes traffic congestion.^22^ Lack of supportive care, cancer care navigators, and transportation services are other barriers leading to health disparities. Lack of universal health insurance predisposes populations to unequal access to cancer care facilities.

### Lack of Novel Technology and Personnel

Practicing in a low-resource country negatively impacts the delivery of quality care. According to a study by Begum et al, there were 632 patients per medical physicist and 549 patients per radiation oncologist in 2009 in Pakistan.^23^ No doubt, the number has declined to one radiation oncologist per 250-300 patients and one radiotherapy technologist per 100-150 patients,^11^ but still not enough to bear this patient burden. Similarly, repair and maintenance personnel increased from 2.11 for every 2 mega voltage units in 2004 to 2.49 in 2009. Hence, physicists, technicians, and oncology nurses, considered middle tier of oncology professionals, are all in short supply to provide high quality of care.^24^

There are currently 57 radiation treatment units in Pakistan; of these, 26 are linear accelerators and 31 are Cobalt-60 machines, averaging <1 unit per 1 million people.^25^ There are 31 radionuclide treatment units and 14 brachytherapy systems in total.^26^ There are a few industry-sponsored trials available at large academic centers, but lack of resources and research funding for investigator-initiated clinical trials in radiation oncology makes latest advancements in the field to be implemented at slow pace in Pakistan.

### Lack of Understanding Among Medical Professionals

In a survey performed among the Pakistani medical students for pursuance of their future career, only 27% wished to accept oncology as their profession.^27^ These students belonged to private medical colleges, had an oncologist, a cancer survivor, or cancer-related death in family, or planned to migrate. The negative perceptions for adopting this career were lack of oncologic facilities in hospitals, radiation exposure, need for private practice, poor prognosis, high patient burden, and depressing environment. A significant proportion of Pakistani physicians migrate to Europe, Middle East, or United States for better training and practicing facilities, leaving insufficient undergraduate trainees in oncology who are not well aware of screening, diagnosis, appropriate referral methods, and more importantly, breaking bad news and communication skills.^28^

### Discrepancy Among Radiation Oncologists for Plan Evaluation and Approval

The complexity of radiation planning and delivery methods makes the radiation treatment vulnerable to errors.^29^ Apart from this, disparities and variations among radiation oncologists in tumor volume delineation can also affect local tumor control and can lead to serious consequences.^30^ These differences exist despite adherence to well-established organ dose criteria and uniform contouring guidelines.^31^ Recently, to overcome this problem, institutes have started the peer review process in Pakistan. Peer review is an important way for quality assurance in treatment planning.^32^ Single-institute analysis of peer review showed that it overcomes deficiencies in radiation treatment plans, with a goal of improved and individualized patient care in Pakistan.^33^ However, there are no resources to communicate with other radiation centers to improve the treatment plans, as most of the centers are not well equipped and do not use electronic medical record for patient information.

### Impact of COVID-19 Pandemic

Since December 2019, the SARS-CoV-2 infection has been a major health issue globally. To overcome the impact of the pandemic, various steps were taken; one is the implementation of telemedicine. Teleclinics have clear advantages to reduce the risk of exposure and protect patients with cancer who are more vulnerable than general population and have worse outcomes if infected.^34^ Although telemedicine was introduced in many Pakistani radiation centers, because of lack of advancement in information technology (IT) and improper internet signals, virtual communication is not of optimal quality. Occasionally, patients also miss their telephonic appointment for follow-up or do not have network coverage in remote areas.

## Steps Toward Mitigating Health Disparity

### Advancement in Technology

In 1947, Pakistan inherited its first Cobalt-60 unit at King Edward University, Lahore, from Britain. After independence, Pakistan Atomic Energy Commission (PAEC) was established in 1956 and it played a pioneering role in the development of radiation therapy and oncology infrastructure.^27^ Gradually, multiple radiation facilities opened and Pakistan started its growth in the field of radiation oncology. Today, Shaukat Khanum Memorial Cancer Hospital and Research Centre (SKMCH and RC), Lahore, is Pakistan's largest cancer hospital.^10^ It gets 50,000 referrals very year, but only 8,000 can be catered for treatment because of finite resources. It has the largest radiation center of Pakistan, with facilities available for interstitial and intracavitary brachytherapy procedures; five newly installed linear accelerators with capability for stereotactic radiosurgery, intensity-modulated radiation therapy, volumetric modulated arc therapy radiation delivery with increased precision, and deep breath-holding technique; two computed tomography simulators; and an Eclipse, a three-dimensional planning system. SKMCH and RC is one of two institutions providing three-dimensional high-dose-rate brachytherapy in Pakistan.

### Collection of Data

The initial step toward addressing the inequality in radiation oncology provision is to quantify the need of radiation technology and personnel that should be enough to fulfill the community requirements, as there are no factual data available on these scarce services. There is an imperative need to reform the undergraduate medical curricula in Pakistan to increase cancer awareness among future physicians.^35^ These aspects have also been emphasized by the Oncology Education Committee of the Cancer Council of Australia.^36^ Central Cancer Registry in Karachi along with the Cancer Control Program^14^ has been launched to establish a centralized cancer database.

### Uniform Access to Health Care

It is vital to provide some sort of universal health coverage at mass level. Provision of health care card by the Sehat Sahulat Program provides a comprehensive service for a variety of medical illnesses including cancer for deserving citizens in an efficient manner. Health insurance is a necessity and not a luxury to improve children's overall health as shown in a study by Aziz et al^37^ in which they showed that health insurance not only helps deserving people but also improves overall health infrastructure in Pakistan.

### Education, Research, and Training

Radiation oncology is a specialty with its own exit examination and fellowship from the College of Physicians and Surgeons, Pakistan. From 2000 to 2013, 29 residents passed the exit examination in radiation oncology. Across the country, about 260 oncologists are registered in all disciplines with postgraduate training practicing clinical oncology. As of 2019, there were about 80 fellows in radiation oncology in good standing with CPSP.^25^ SKMCH and RC has the largest residency and fellowship training program accredited for radiation oncology training by CPSP, currently enrolling 12 residents, two fellow doctors, and one senior instructor. The curriculum of residency program includes daily lectures and didactics, weekly peer review and quality assurance meetings, courses on radiation protection, basic life support, advanced cardiac life support, workshops on communication skills, research methodology and dissertation writing, and annual symposium. Research work is an integral part of the residency program. Researchers from the institution have contributed more than 465 research articles that are published in renowned indexed journals. It is staffed by radiation oncologists trained at the best cancer centers in the United Kingdom, Canada, and the United States; fully trained radiation technologists; medical physicists; oncology nurses; and radiation oncology house staff. Every year, there is a rise in the trend in treated number of patients by surgeons and radiation and medical oncologists.^10^ About 75% of the patients are currently financially assisted by SKMCH and RC.^25^ SKMCH and RC is now expanding and has constructed one hospital in Peshawar, and a third comprehensive cancer hospital in Karachi is in progress, with architectural renderings finalized and construction underway. All these measures are taken to decrease health disparities in Pakistan.

PAEC is also worth mentioning as 19 cancer centers are working under its supervision. Initially, it primarily focused on nuclear medicine. They gradually developed more projects and are currently working on five major departments (nuclear medicine, cancer hospitals, research and development center, agriculture, and biotechnology and engineering department). Their work in the field of oncology started from radiation therapy with introduction of medical oncology later on, but surgical and palliative care units are yet to be established. According to the survey by Begum et al,^23^ the newly registered patients in all the PAEC cancer centers raised from 33,000 in 2005 to 46,000 in 2009. PAEC is therefore installing more equipment to cope with cancer burden and has also established its cancer registry^38^ for improving the cancer care delivery. Other institutions in private sectors like Agha Khan University Hospital, Karachi, and Shifa International Hospital, Islamabad, have robust radiation oncology departments providing quality cancer care to patients.

In conclusion, lack of education, cultural barriers, logistic issues, skilled personnel and technology underdevelopment, insufficient understanding among medical professionals, discrepancy among radiation oncologists regarding plan evaluation, and negative COVID-19 impact are some of the factors contributing to radiation oncology health disparities in Pakistan.

Better coordination with other radiation therapy centers, free online journal access, standardizing peer review process, and cost-effective education for staff with the help of telecommunication are important steps taken toward improved quality of radiation oncology care in Pakistan. Radiation oncologists in particular can now take advantage of the global telecommunication technology explosion, and in response to the challenge of the fight against cancer, greater collaborative global cancer care effort is needed in both research and education. Although the path is difficult with perceivable health inequalities in radiation oncology care in Pakistan, there is an immense potential for improvement in foreseeable future with strategically chosen pathways in collaboration with the public-private sector.
